# The effect of sport for LIFE: all island in children from low socio-economic status: a clustered randomized controlled trial

**DOI:** 10.1186/s12955-019-1133-x

**Published:** 2019-04-16

**Authors:** Gavin Breslin, Stephen Shannon, Ruth Rafferty, Ben Fitzpatrick, Sarahjane Belton, Wesley O’Brien, Fiona C. Chambers, Tandy Haughey, Donncha Hanna, Richard Gormley, Darryl McCullagh, Deirdre Brennan

**Affiliations:** 1 Sport and Exercise Sciences Research Institute, Ulster University, Jordanstown, Newtownabbey, Northern Ireland, UK; 2 School of Health and Human Performance, Dublin City University, Dublin, Ireland; 3 School of Education, Sports Studies and Physical Education Department, University College Cork, Cork, Ireland; 4 School of Psychology, Queen’s University Belfast, Belfast, Northern Ireland, UK

## Abstract

**Background:**

School-based interventions offer the opportunity to increase physical activity, health-related quality of life (HRQOL) and nutritional behaviours, yet methodological limitations hinder current research, particularly among under-represented children from low socio-economic status (SES). The aim was to determine the effect of a 12-week physical activity programme, Sport for LIFE: All Island (SFL:AI), on physical activity levels, HRQOL, and nutritional attitudes and behaviours in children of low SES across the island of Ireland.

**Methods:**

A 2 (groups) × 4 (data collection points) clustered randomised controlled trial was conducted comprising an intervention group who received SFL:AI for 12 weeks, and a waiting-list control condition. In total 740 children (381 boys, 359 girls) aged 8–9 years (mean = 8.7; SD = .50) from 27 schools across four regions of Ireland (Ulster, Leinster, Connacht and Munster) took part. Physical activity was measured by accelerometers, and children completed a validated questionnaire at baseline, mid (i.e. 6-weeks), post-intervention (i.e. 12 weeks) and follow-up (i.e. 3 months post-intervention).

**Results:**

No significant interaction effects for the intervention were found on any of the study outcomes. Main effects were reported for physical well-being, parental relations and autonomy and financial resources, as well as sweetened beverages, environment and intake, and attitude to vegetables. However, these changes were not statistically attributable to the intervention.

**Conclusions:**

It remains unclear if school-based physical activity interventions can improve HRQOL through physical activity with children from low SES. Logistical and methodological considerations are outlined to explore the null effect of the programme, and to provide suggestions for future research and practice.

**Trial registration:**

Trial registration number: ISRCTN76261698.

Name of registry: ICRCTN.

Date of registration: 23/08/2017.

Date of enrolment: September 2014.

## Introduction

Health-related quality of life (HRQOL) is a multi-dimensional construct and refers to physical, psychological, social and behavioural components of children’s well-being [1]. Regular participation in physical activity is associated with better HRQOL in children [1, 2]. Current guidelines advise that all children should engage in at least 60 min of moderate-to-vigorous intensity physical activity (MVPA) everyday [3, 4]. Indeed, children who met the recommended guidelines of MVPA score higher on multiple HRQOL domains than less active children [5–8]. Yet, physical activity prevalence data shows that most children are insufficiently active. Currently, 24% of pre-adolescent children in Northern Ireland [9] and 8.6% of boys and 2.9% of girls aged 9 years in England [10] are insufficiently active. These statistics are reflected in data from 122 countries [11] that approximately 80% of school youth do not meet the MVPA guidelines for health. Further, longitudinal evidence suggests that physical activity behaviours developed during childhood decline during adolescence and adulthood [12], and furthermore, children of low socio-economic status (SES) are at a higher risk of inactivity, and developing subsequent health problems [13]. Such evidence highlights the need to promote physical activity at an early age in order to curb the declining rates.

### School-based physical activity interventions

The development of effective physical activity interventions is a key public health target, and many interventions have been implemented and evaluated [14, 15]. There are a significant number of school-based physical activity interventions for children [16], with the benefit that school attendance is compulsory and children spend most of their waking hours at school [17], and there is less chance of poor adherence to the intervention [18]. Indeed, school-based physical activity interventions can reach those children from areas of low SES that otherwise may have not had leisure opportunities [18]. Systematic reviews examining the effect of school-based physical activity interventions reported that most were effective, albeit at a small-to-moderate level for increasing physical activity [14, 16, 19]. Furthermore, theory-driven physical activity interventions that were multicomponent (i.e. targeted a healthy lifestyle) were found to be most effective [14]. These findings are reflected in the World Health Organization’s recommendations on physical activity and diet interventions [20]. Limitations of such studies have been reported however [14], including: a lack of valid physical activity measurements, few studies with long-term follow-up, and limited reporting of randomization procedures [14], outlining the need for further rigorous research. In addition, while school-based interventions have shown some success at improving aspects of children’s physical health, such as body mass index [21, 22] the evidence for children’s HRQOL is less clear [23], as outlined below.

### School based physical activity interventions for HRQOL promotion

Systematic reviews have reported a small inverse association between physical activity, depression and anxiety [24] and a moderate effect size for self-esteem, at least in the short term [25]. Recently, a systematic review of the effect of school-based physical activity programmes on children’s HRQOL found that three of eleven studies increased components of children’s well-being [23], but had low quality research designs [1] and measurement inconsistencies [26]. For instance, randomised designs were infrequent, and most positive intervention effects were found on single well-being dimensions (i.e. psychological well-being), which do not capture a multi-dimensional well-being concept such as HRQOL. The authors concluded that future studies should apply a multi-dimensional child-centred well-being measure, such as HRQOL, and an objective measure of physical activity [23] in randomised intervention designs.

### Sport for LIFE: All Island intervention Programme

The current intervention, Sport for LIFE:AI (Sport is for Living, Integration, Fun and Education: All Island), was based on a previous successful Sport for LIFE programme that was conducted from 2010 to 2011 with children from low SES in Northern Ireland [27]. Sport for LIFE is a 12-week physical activity and healthy eating programme designed to promote an active lifestyle and the importance of a healthy balanced diet [27]. It was based on other effective school-based physical activity and nutritional interventions [21] and designed using Social Cognitive Theory (SCT) [28] as a framework to develop the programme’s content. The intervention was effective at significantly increasing physical activity, in addition to improving nutritional attitudes and behaviours [27].

Given replication is a cornerstone of the scientific method, and replication of previously found successful interventions is an area much lacking in public health efforts [15, 20], The current study using Sport for LIFE:AI sought to increase the reach of SFL and emulate its previous success by targeting a larger sample of 4000 children in low SES areas across the four provinces of Ireland (Ulster, Munster, Leinster and Connacht), and delivered in partnership with a further four third level academic institutions across Ireland. The research aspect of programme was enhanced to address the criticisms of previous school-based studies lasting less than 3 months [14], by adopting a longitudinal clustered randomised controlled design. In addition, the inconsistency in the measurement of well-being [23] and physical activity [19] in school-based interventions was addressed by using a multi-dimensional measure of HRQOL developed from a child’s perspective (i.e. Kidscreen-27) and wearable technologies measuring physical activity. Sport for LIFE:AI is the first ‘All island’ (Northern Ireland and Republic of Ireland) collaboration between academic institutions aimed at promoting physical activity to children of low SES, and the present study addressed some of the logistical and operational challenges of conducting a large-scale intervention.

### Aims and hypotheses

To determine whether a theory-driven multicomponent Sport for LIFE:AI programme improved physical activity, HRQOL and nutritional attitudes and behaviours in 8–9-year-old children from low SES across Ireland. The study also aimed to discuss some of the challenges associated with implementing a large scale nationwide physical activity intervention in schools with children in areas of low SES. Based on the previous Sport For LIFE findings [27] it was hypothesised that the intervention group would significantly increase their physical activity levels and nutritional attitudes and behaviours in comparison to the control group. Secondly, it was hypothesised that a significant increase in physical activity would cause a concurrent improvement in the HRQOL of the intervention group in comparison to the control.

## Methods

### Study design

A 2 (groups) × 4 (data collection points) clustered randomised controlled trial was conducted with primary school children aged 8–9 years of low SES. The study followed the Consolidated Standards of Reporting Trials (CONSORT) guidelines [29]. The study protocol was published on the International Standard Randomized Controlled Trial Number (ISRCTN) registry (protocol number: ISRCTN76261698). Schools across the four regions of Ireland (Ulster, Leinster, Munster and Connacht) participated in the research and data was collected at baseline (week 0), mid-point (week 6), post-intervention (week 13) and at follow-up (6 months post-intervention). The intervention group received the SFL:AI intervention for 12 weeks; the control group continued with their regular school day activities and received the intervention at a later date.

### Schools and participants

Primary schools across the four regions (i.e. Ulster, Munster, Leinster and Connacht) of Ireland in two jurisdictions (i.e. Northern Ireland and Republic of Ireland) were invited to participate. In Northern Ireland, schools were identified using the Multiple Deprivation Measure [30] that ranks areas based on seven domains of deprivation: income, employment, health, education, proximity to services, living environment and crime. Schools situated in the most deprived areas were identified. In the Republic of Ireland schools identified by the Department of Education’s Delivering Equality of Opportunity in Schools (DEIS) programme were included [31]. The criteria for a DEIS school included: lone parenthood, local authority accommodation, Travellers, large families (five or more children) and pupils eligible for free books.

For research purposes, two levels of school inclusion criteria were also included to protect against potential confounders [32]. These were (1) schools must have a sports hall; and (2) they must be co-educational (include both genders) or have a comparative number of participants in a similar single-gender school. A total of 98 schools were invited to participate, of which 27 selected schools were randomly assigned to the intervention group (*n* = 14) and the control group (*n* = 13) by three members of the research team using a manual random number generator. One researcher placed a school’s name in a closed envelope. A second researcher shuffled the envelopes. Blinded to this procedure, a third researcher then selected an envelope representing a school and coded it as intervention or control. It was not possible to blind schools to their allocation as agreement was sought for the delivery, or waited delivery of the intervention prior to data collection.

Recruitment began after ethical approval was obtained from the Ulster University Research Ethics Committee. Children from Year 5 (3rd class in Republic of Ireland) and their parents provided assent and consent respectively to participate. Using G*POWER for mixed Analysis of Variance for two groups across four time points, where *p* = 0.05, *f* = 0.05, and a Power of 80%, a G*POWER calculation (Institute for Digital Research and Education Software, 2015) yielded a minimum total sample size of 644 participants. Taking an attrition rate of 19% into consideration (observed in Breslin et al., 2012), the intended sample size was 766 children. For clustering the sample, a total of 30 schools (control = 15, intervention = 15) were targeted based on the mean number of recruited children per school (*n* = 26, recorded in Breslin et al., 2012).

### Physical activity intervention programme

This SFL:AI programme was the same as delivered in Northern Ireland in 2012 [19, 27]. The 12 weekly Sport for LIFE:AI physical activity sessions were underpinned by components of Bandura’s Social Cognitive Theory [28] which included goal-setting, problem solving and self-monitoring. Each of the 12 week lessons were based on a specific educational theme that tied into the Northern Ireland Key Stage 2 Curriculum [33] and the World Health Organization’s School Policy Framework [34] on promoting health in schools (see Breslin et al. [27] for detailed information on the lessons included in the programme).

Intervention schools in the Ulster and Leinster regions received the programme from September–December 2014; the control schools received the programme from September–December in 2015. Intervention schools in the Connacht and Munster regions received the programme from January–March 2015; the control schools received the programme from September–December 2015. Despite receiving the programme later, the control schools took part in the research at the same time as the intervention schools to aid comparisons. The intervention schools were advised by the researchers to continue teaching their standard physical education curriculum alongside the programme while the control groups were asked to continue their physical education classes as normal.

In order for the programme to be delivered to the 27 schools across the four provinces of the island of Ireland it was necessary to recruit student volunteers at each of the five participating institutions (Ulster University, Dublin City University, Sligo Institute of Technology, Galway and Mayo Institute of Technology, University College Cork) situated across the provinces. All volunteers were given standardised training of the 12 lessons in SFL:AI at each of the participating institutions in Ireland by a senior researcher. Each lesson was delivered in the school sports hall using the basic sports equipment provided by the SFL:AI project. Throughout each lesson children were provided activities intended to get them to achieve moderate-to-vigorous intensity (MVPA) level for enhancing health [35]. In addition, teachers were provided with weekly teaching resources, an educational DVD of the programme and a SFL:AI t-shirt.

### Procedure

**Fig. 1:**
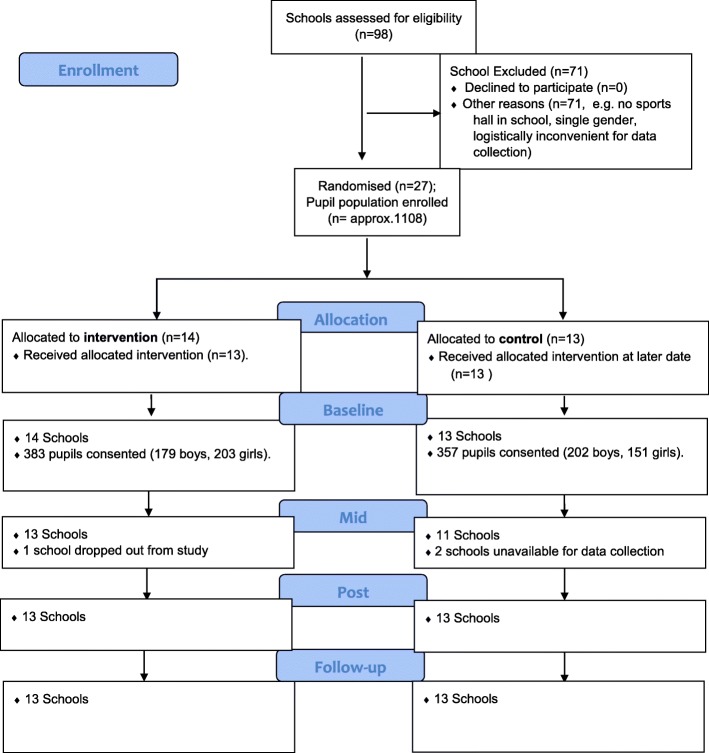
CONSORT Flow Diagram of Participants

A trained research team from each University conducted data collection across the four regions. During fieldwork children were given accelerometers, with clear instructions on their use, during class time while supervised by the research team and teacher. Accelerometers were initialised prior to each test and the data retrieved after 8 days using Actilife software (v6 USA California, AM 7164–2.2 by MTI Health Services, FL 32548, USA). Children were asked to self-report items in a questionnaire containing HRQOL and nutrition scales, in which a trained researcher adopting a neural demeanour and accompanied children in groups of 5–10 to provide support for interpretation of the questionnaire. Figure 1 details the staggered delivery of the intervention and accompanying data collection.

### Outcome measures

#### Total and moderate-to-vigorous physical activity (MVPA)

Daily total and moderate-to-vigorous physical activity (MVPA) was measured using Actigraph GT3x accelerometers (Actigraph, GT3X California, AM 7164–2.2 by MTI Health Services, Fort Walton Beach, FL 32548, USA) in a subsample of children at baseline, post-intervention and follow-up. The accelerometers measured the frequency, intensity and duration of activity levels, and are valid and reliable [36]. Participants were asked to wear the accelerometer for 8 days at the midaxillary line above the right hip on an elasticated band, removing it for water-based activities and bed-time. The wear-time criteria used in the previous Sport for LIFE study with children of the same age and SES [19, 27] was used to ensure results were comparable. Due to the sporadic nature of children’s activity patterns, a five-second epoch was chosen [37]. Mattock’s physical activity cut-off points were used to calculate time spent in light, moderate and vigorous physical activity [38]. The actigraph data was then processed using Actilife software.

### HRQOL

The KIDSCREEN-27 instrument measured HRQOL, and is validated for children aged 8–18 years [39]. Although the original development of KIDSCREEN-27 measured HRQOL across five dimensions [39], a recent confirmatory factor analysis of the self-report measure with children aged 8–9 years from low SES found that a 7-factor structure was a better fit for this population [40]. These included: Physical well-being (5 items); Psychological well-being (4 items); Moods and emotions (3 items); Autonomy and parent relations (5 items); Financial resources (2 items); Social support and peers (4 items) and School environment (4 items). A 5-point Likert scale was used to measure the frequency of feelings or behaviours (1 = never, 2 = seldom, 3 = sometimes, 4 = often, 5 = always) or the intensity of an attitude (1 = not at all, 2 = slightly, 3 = moderately, 4 = very, 5 = extremely), all referring to the previous week. KIDSCREEN-27 also measures a total score for HRQOL, with higher scores indicating better HRQOL. HRQOL was measured at all time-points.

### Nutrition

Children’s dietary patterns, attitudes, behaviours and environment associated with healthy eating were assessed at all time-points using an Australian Child Nutrition Questionnaire [41] which was slightly modified to reflect foods and beverages commonly found across Ireland. This questionnaire was used previously in the Sport For LIFE research study in 2012 [19, 27]. The questionnaire assessed food intake (six items: non-core foods, fruit, vegetables, water, sweetened beverages and sweetened beverages without diet drinks), healthy eating behaviour (one item), nutrition attitudes (two items: attitudes towards fruit, attitudes towards vegetables) and environment (one item, home or school environment fruit and vegetables are consumed). Higher scores represent increased intake or a better attitude/environment.

### Statistical analysis

All data met the assumptions for parametric statistical analysis. Descriptive statistics (mean and standard deviation) were calculated across all time-points for minutes spent in MVPA, total physical activity, total score of KIDSCREEN-27, and each dimension of KIDSCREEN-27. General linear models with repeated measures were used to compare the intervention and control groups’ mean scores across each of the time-points. Where Mauchly’s Test of Sphericity was violated, the Green-house Geisser value was reported.

Changes in objectively measured MVPA and total physical activity were analysed separately with 2 × 3 repeated measures analysis of variance (ANOVA) with three time-points (baseline, post-intervention and follow-up) and group (intervention and control) as the independent variables and MVPA and total physical activity as the dependent variables for each group. As physical activity data was collected at different times of the year (corresponding with the delivery of the intervention across the island), analysis was adjusted to include season as a categorical covariate (i.e., winter, spring, summer and autumn) to control for the potential seasonal effect on physical activity levels [42].

To investigate changes in HRQOL and nutritional sub-scales, separate 2 × 4 repeated measures mixed analysis of variance (ANOVA) were used with testing session (baseline, mid-point, post-intervention and follow-up) and group (intervention and control) as the independent variables and total well-being, each HRQOL dimension and each nutrition sub-scale as the dependent variables for each group. A significance level of *p* < .05 was adhered to and partial eta squared (ηp2) was calculated as a measure of effect size, with .01, .06 or .14 or above considered as small, medium or large effect sizes respectively [43]. All analyses were computed using the Statistical Package for the Social Sciences (SPSS V.22).

## Results

A total of 98 schools eligibility were assessed with 27 schools invited and agreeing to participate (see Fig. 1 CONSORT flow diagram). Following randomization, 14 schools were allocated to the intervention group, and 13 were allocated as wait-list control. Due to a student volunteer being unavailable to deliver the programme, one intervention and three control schools were unable to be included in the analyses. Further practical challenges regarding the implementation of the intervention are reported in a subsection of the discussion.

The total sample size was 740 children (381 boys, 359 girls, mean age = 8.7 years (SD = 0.5). The number of boys and girls in each group across each region of Ireland is shown in Table 1. On average, 12% attrition was found across the measurement points, based on the number of children who completed the self-report measurements. Chi-square tests confirmed no significant differences between the intervention and control group for the number of children who completed measurements at each time-point.

**Table 1:** The total number of children for each group, gender and region

	Intervention	Control	Ulster	Leinster	Munster	Connacht
Male	179	202	89	103	115	74
Female	203	151	107	81	85	86
Total	383	357	196	184	200	160

### Accelerometer assessed physical activity

From the 408 children given accelerometers at baseline, 224 children (54.9%) met the wear-time criteria. At post-intervention 339 children wore an accelerometer and 77 children (22.7%) met the criteria, while at follow-up 194 children wore an accelerometer and 57 (29%) met the criteria.

Table 2 shows the mean and standard deviation scores and statistical analyses for physical activity and HRQOL for the intervention and control groups. The change in MVPA and total physical activity in each group from baseline to follow-up was analysed by a repeated measures ANOVA with three time-points (baseline, post-intervention and follow-up). There were no significant main effects for time or group and no significant interaction effect for the intervention for MVPA and total physical activity.

**Table 2:** Descriptive and statistical analyses with repeated measures by study group for physical activity and well-being

Variables	Intervention M (n); SD	Control M(n); SD	Time F (p)	Time^a^Group F(p); Np^2^
Accelerometer - MVPA				
Baseline	35.86 (125); 16.10	34.14 (99); 12.70		
Post	33.14 (50); 13.06	31.04 (27) 13.05	.580 (*p* = .56)	.562 (*p* = .57); .04
Follow-up	37.53 (30); 15.58	37.65 (27); 13.55		
Accelerometer- Total PA				
Baseline	266.41 (125); 48.82	257.13 (99); 43.48		
Post	268.02 (50); 56.23	257.47 (27); 59.88	.574 (*p*=. 57)	.251 (*p* = 78); .05
Follow up	275.77 (33); 56.25	274.86 (27); 43.67		
K-27 Total				
Baseline	115.91 (273); 11.72	114.6 (273); 12.15		
Mid	116.24 (282); 14.32	116.48 (250) 13.58		
Post	115.58 (250); 14.60	115.89 (271);12.66	1.380 (*p* = .24)	.434 (*p* = .71); .00
Follow-up	116.38 (251); 12.68	116.00 (236);12.11		
K-27 Physical well-being				
Baseline	19.40 (334); 2.03	19.52 (314); 2.89		
Mid	20.08 (304); 2.9	19.81 (285); 2.98	35.043 (*p* = .00)	1.491 (*p* = .21); .00
Post	18.80 (294); 2.61	18.83 (309); 2.28		
Follow-up	18.76 (291); 2.41	18.69 (260); 2.64		
K-27 Psychological well-being				
Baseline	18.06 (338); 2.38	18.06 (315); 2.50		
Mid	17.97 (319); 2.74	18.09 (281); 2.40	.554 (*p* = .64)	.393 (*p* = .75); .00
Post	18.12 (300); 2.61	18.31 (316); 2.23		
Follow-up	18.09 (97); 2.49	18.20 (272); 2.18		
K-27 Moods & emotions				
Baseline	13.23 (343); 2.23	13.07 (329); 2.26		
Mid	13.16 (32); 2.39	13.28 (288); 2.13	.638 (*p* = .59)	3.642 (*p* = .01); .009
Post	13.04 (303); 2.46	13.36 (326); 2.26		
Follow-up	13.15 (303); 2.20	13.48 (277); 1.83		
K-27 Parents & autonomy				
Baseline	21.37 (33); 3.46	21.13 (318); 3.51		
Mid	21.41 (319); 4.29	21.55 (287); 3.74	5.608 (*p* = .01)	.764 (*p* = .51); 002
Post	21.88 (298); 4.03	21.76 (322); 3.74		
Follow-up	21.96 (296); 3.58	22.00 (274); 3.13		
K-27 Finance				
Baseline	7.48 (351); 2.42	7.26 (334); 2.48		
Mid	7.60 (324); 2.38	7.63 (290); 2.47	4.214 (*p* = .00)	.120 (*p* = .94); 00
Post	7.71 (308); 2.31	7.66 (318); 2.44		
Follow-up	7.85 (306); 2.18	7.89 (276); 2.17		
K-27 Social support & peers				
Baseline	17.91 (345); 3.01	17.82 (331); 2.87		
Mid	17.72 (320); 3.07	17.97 (288); 2.95	.801 (*p* = .49)	.225 (*p* = .87); .00
Post	17.80 (305); 3.15	18.14 (317); 2.57		
Follow-up	18.10 (301); 2.70	18.15 (270); 2.57		
K-27 School environment				
Baseline	17.63 (346); 2.87	17.23 (325); 3.10		
Mid	17.86 (319); 2.88	17,36 (285); 3.20	2.633 (*p* = .051)	.341 (*p* = .79); .00
Post	17.93 (304); 2.98	17.67 (316); 3.11		
Follow-up	17.67 (299); 3.04	17.44 (272); 3.00		

Note^a^ M = mean; n = sample size; SD = standard deviation; K-27 = Kidscreen-27; p = probability value; Np^2^ = effect size

### Well-being

Green-house Geisser was reported for each model as sphericity was not assumed. A significant main effect for time (F (2,549) = 35.043, *p* < .01, ηp2 = .009) was reported for physical well-being. Planned contrasts revealed that the significant within-group changes were attributable to a small decrease in both groups’ mean scores at 3 months follow-up in comparison to their baseline and mid-point scores. No main effect for group or interaction effect was found. A significant interaction effect for moods and emotions was reported (F (2, 578) = 3.642, *p* < .01, η_p_^2^ = .009). Planned contrasts showed that the control groups’ mean score increased from 13.09 (SD = 2.39) at baseline to 13.61 (SD = 1.78) at 3 months follow-up, while the intervention groups’ mean score marginally decreased from 13.38 (SD = 2.15) at baseline to 13.11 (SD = 2.23) at 3 months follow-up. No main effect for group or time was found. A significant main effect for time was reported for parental relations and autonomy (F (2, 568) = 5.608, *p* < .01, η_p_^2^ = .002), but no significant main effect for group or interaction effect for the intervention was found. Planned contrasts revealed that both groups’ mean scores were significantly increased at 3 months follow up in comparison to their baseline and mid-point scores. Finally, a significant main effect for time was reported for financial resources (F (2, 580) =4.214, *p* < .01, η_p_^2^ = .01) but no significant main effect for group or interaction effect for the intervention was found. Planned contrasts revealed that both groups’ mean scores were significantly increased at 3 months follow up in comparison to their baseline. No significant main effect for group or time or interaction effect was reported for any of the other KIDSCREEN-27 variables.

### Nutritional attitudes and behaviours

Table 3 shows the mean and standard deviation scores and statistical analyses for nutrition for the intervention and control groups. A significant main effect for time was reported for vegetable intake (F (2.91, 1089.5) = 6.177, *p* < .001, η_p_2 = .016), attitude towards vegetables (F(1.65,653.58) = 494.2, *p* < .001, η_p_^2^ = .55), sweetened beverage (F(2.9703) = 3.39, *p* < .05, η_p_^2^ = .014) and environment (F(1.84,601.2) = 896.6, *p* < .001, η_p_^2^ = .73) but no significant main effect for group or interaction effect for the intervention was found.

**Table 3:** Descriptive and statistical analyses with repeated measures by study group for nutrition sub-scales

Variables	Intervention M (n); SD	Control M(n); SD	Time F (p)	Time^a^Group F(p); Np^2^
Non-core food intake				
Baseline	8.94 (119); 4.9	8.37 (111); 5.04		
Mid	8.24 (119); 5.5	8.04 (111) 5.94	2.45 (*p* = .063)	1.35 (*p* = .256); .006
Post	8.24 (119); 5.48	7.64 (111); 5.4		
Follow-up	8.54 (119); 5.84	7.0 (111); 5.3		
Sweetened beverage intake				
Baseline	4.56 (130); 2.63	4.39 (114); 2.53		
Mid	4.8 (130); 2.95	4.44 (114); 3.43	3.391 (*p* = .019)	1.178 (*p* = .317); .005
Post	4.5 (130); 2.97	3.93 (114); 2.88		
Follow-up	4.5 (130); 3.02	3.63 (114); 2.73		
Water intake				
Baseline	4.44 (203); 1.49	4.26 (179); 1.62		
Mid	4.52 (203); 1.29	4.44 (179); 1.51	1.248 (*p* = .29)	1.167 (*p* = .32); .00
Post	4.38 (203); 1.45	4.47 (179); 1.34		
Follow-up	4.46 (203); 1.41	4.48 (179); 1.42		
Fruit intake				
Baseline	3.97 (199); 1.82	3.98 (184); 1.65		
Mid	3.91 (199); 1.8	3.91 (184); 1.65	.684 (*p* = .56)	0.067 (*p* = .978); .00
Post	3.97 (199); 1.79	3.89 (184); 1.6		
Follow-up	4.06 (199); 1.69	4.03 (184); 1.58		
Vegetable intake				
Baseline	2.69 (194); 1.56	2.77 (182); 1.55		
Mid	2.85 (194); 1.6	2.82 (182); 1.59	6.177 (*p* = .000)	.817 (*p* = .48); 002
Post	2.85 (194); 1.55	3.04 (182); 1.46		
Follow-up	2.97 (194); 1.57	3.16 (182); 1.43		
Healthy Behaviour				
Baseline	13.36 (121); 4.2	14.17 (112); 3.84		
Mid	13.66 (121); 4.4	14.0 (112); 3.34	1.115 (*p* = .341)	.458 (*p* = .703); .002
Post	13.68 (121); 4.51	14.22 (112); 3.66		
Follow-up	13.77 (121); 4.03	14.58 (112); 3.7		
Attitude towards fruit				
Baseline	18.13 (214); 3.08	18.37 (190); 2.37		
Mid	18.44 (214); 2.71	18.32 (190); 2.41		
Post	18.35 (214); 2.69	18.66 (190); 2.16	1.61 (*p* = .187)	1.37 (*p* = .25); .003
Follow-up	18.21 (214); 2.68	18.47 (190); 2.23		
Attitude towards vegetable				
Baseline	16.05 (211); 3.78	16.0 (187); 3.81		
Mid	15.98 (211); 3.94	15.86 (187); 3.8	4.94 (*p* = .000)	.154 (*p* = .82); .000
Post	16.39 (211); 3.71	16.44 (187); 3.89		
Follow-up	16.22 (211); 3.61	16.45 (187); 3.34		
Environment				
Baseline	18.74 (167); 3.9	19.24 (161); 3.28		
Mid	18.97 (167); 3.49	19.39 (161); 3.25	896.6 (*p* = .000)	1.57 (*p* = .21); .005
Post	19.03 (167); 3.78	19.76 (161); 3.18		
Follow-up	19.32 (167); 3.25	19.8 (161); 3.12		

Note^a^ M = mean; n = sample size; SD = standard deviation; K-27 = Kidscreen-27; p = probability value; Np^2^ = effect size

Planned contrasts revealed that both groups’ mean scores were significantly increased at post-intervention and 3 months follow up in comparison to their baseline scores and mid-point scores for vegetable intake. In addition, for attitude towards vegetables, planned contrasts revealed that both groups’ mean scores were significantly increased at post-intervention and follow-up in comparison to their baseline and mid-point scores. Finally, planned contrasts revealed that both groups’ sweetened beverage mean scores were significantly decreased at post-intervention and follow up in comparison to their mid-point scores. No further main or interaction effects were observed for any other subscales.

## Discussion

Despite the predictions and findings reported previously for the Sport For LIFE programme there was no significant effect of the programme on physical activity, HRQOL or nutritional attitudes and behaviours for the intervention group in comparison to the control group. The significant within-groups effects found for the HRQOL and nutritional factors (discussed below) occurred when baseline scores were compared to 3 months follow-up, suggesting that the intervention did not influence these changes. While these findings support the growing view that modest school-based programmes are unlikely to substantially alter children’s daily MVPA or HRQOL [44] there are potential explanations that may reveal why there were null effects, and offer solutions for how future research and practice can aim to increase physical activity and positively influence the health of children from low SES.

The programme did not significantly increase total physical activity or MVPA in children from baseline, post-intervention and 3 months follow-up, which was unexpected. There are a number of possible reasons for the lack of significance in this study. For instance, studies on the effect of school-based physical activity interventions reported that the involvement of families and school staff (e.g. teachers, classroom assistants) within school-based physical activity interventions has been shown to be efficacious [19, 45–47]. In the Sport for LIFE: AI programme, parents were not actively involved and school staff in general were not an integral part of the intervention as it was delivered by a student volunteer, and such limitations may explain why children’s physical activity levels did not significantly change. Encouraging teachers, parents and school staff to role model or become actively involved in encouraging physical activity behaviours may enhance behaviour change in children [48]. Furthermore, measuring the physical activity environment (e.g. motivational climate) may have improved our understanding of the null effects.

Likewise, no significant intervention effect was found for nutritional attitudes or eating behaviours, measured by the same questionnaire used in the previous Sport for LIFE study [25]. It is possible that the nutrition lessons, of which there are two out of the twelve, were not enough to influence nutritional changes in children from low SES. In addition, children’s attitudes and behaviours towards nutrition may be influenced more during home life. Hence, parental involvement in this component of the intervention may have helped change children’s dietary patterns and attitudes [48]. Given there was no intervention effect for physical activity levels, it was unsurprising that HRQOL did not significantly improve. It is proposed that to improve HRQOL through physical activity, interventions may target social (e.g. perceptions of support) and psychological factors (e.g. motivation), and as such may be considered in future programme design.

Beyond the above recommendations, a possible limitation of this study was the lack of a comprehensive process evaluation conducted during the intervention, that help researchers determine adherence to the intervention, practical challenges and participant engagement [49]. Despite uniform training of student volunteers in the delivery of the programme, it is possible that there was varied delivery of the intervention across schools/regions, maybe due to practical, logistical and weather challenges [49]. Further, despite schools being asked not to replace their physical education lesson with Sport for LIFE:AI sessions (ie., they should have been in addition to regular PE lessons), feedback from student volunteers indicated that SFL:AI were used as replacement sessions, not additional sessions. Indeed, research with Irish school teachers has demonstrated that teachers experience barriers to the integration of physical activity into their teaching time, such as limited time and insufficient space [50], and the replacement of physical education with the SFL:AI may reflect a lack of emphasis by education authorities with regards to promoting a culture of physical activity [51]. Anecdotal evidence also revealed that many of the control schools took up the opportunity to engage with other freely available sport programmes such as those provided by other organizations (e.g. Gaelic Athletic Association, Irish football Association). This suggests that it may have been difficult to detect any significant changes in physical activity between the groups. As it would be unfair to ask schools in areas of social and economic disadvantage to abstain from programmes from other sports organisations, future research should record for the number of activity sessions children attend, in addition to participation in other programmes.

From a measurement perspective, accelerometer compliance was necessary to accurately capture physical activity levels. However, from the 408 children given an accelerometer at baseline, only 57 provided valid data at follow-up measurements. Indeed, recent research indicates that participants are unlikely to adhere to stringent accelerometer wear-time criteria across several data collection points [52]. A strategy for compliance is giving the children an activity diary to complete (with the help of their parents) or asking the teacher to also wear an accelerometer to act as a role model [53]. Such strategies were not implemented in SFL:AI because of the difficulty in patrolling the diaries, and lack of device accessibly. Indeed, the logistics and finite resources of accelerometers and personnel did not enable measurements at each time point to be conducted simultaneously. It has been suggested that staggered data collection on such a large sample in this study compared to the previous SFL programme may compromise data comparability and study power due to possible diverse weather conditions, differential monitor reactivity, and other important school events (e.g. parties) which can lead to unwanted group differences [49]. Therefore, in order to minimise the impact of unwanted group differences, simultaneous data collection in all study groups should be considered [54]. Moreover, due to logistical challenges associated with downloading and calibration of accelerometers, accelerometers were not used at time point 2 (6 weeks into the programme), which we speculate may have been the period where children were more active.

Despite the above limitations of the study that have been addressed, the strengths of the study were testing a physical activity intervention with a longitudinal clustered randomised design, use of objective measures of physical activity and a multi-dimensional measure of HRQOL developed from children’s perspectives (KIDSCREEN-27), and reporting the study’s findings in line with the CONSORT guidelines, which demonstrated a level of transparency in the research process. Sport for LIFE:AI was the first school-based physical activity intervention to be studied across both jurisdictions on the island of Ireland and also collaborated with by involving other third level academic institutions across Ireland. As a first of its kind study, it is envisaged that the lessons learned from this research will be used to continue research of this kind across both jurisdictions.

## Conclusions

In conclusion, Sport for LIFE:AI did not significantly improve physical activity, HRQOL or nutrition behaviours of children aged 8–9 years from low SES. Despite the lack of significant findings, some recommendations have been suggested for future programme improvements in large scale studies. Specifically, inclusion of parents and school staff into the intervention as role models that provide continued encouragement are needed. Process evaluations should be conducted to help researchers understand the fidelity of an intervention regarding what improvements could be made to ensure effective and efficient delivery [48] and further research is needed to determine what strategies are most effective for accelerometer compliance with children from low SES. These additions may have an impact on increasing physical activity, and its role in children’s HRQOL and nutritional attitudes and behaviours.

## Abbreviations

HRQOL: Health-related quality of life
SES: Socio-economic status
SFL:AI: Sport for LIFE: All Island
WHO: World Health Organisations
